# Measuring substance use in the club setting: a feasibility study using biochemical markers

**DOI:** 10.1186/1747-597X-7-7

**Published:** 2012-02-09

**Authors:** Johanna Gripenberg-Abdon, Tobias H Elgán, Eva Wallin, Marjan Shaafati, Olof Beck, Sven Andréasson

**Affiliations:** 1 Department of Public Health Sciences, Karolinska Institutet, Stockholm, Sweden; 2 STAD, Stockholm Centre for Psychiatric Research and Education, Department of Clinical Neuroscience, Karolinska Institutet and Stockholm County Council Health Care Provision, Stockholm, Sweden; 3 Department of Medicine, Section of Clinical Pharmacology, Karolinska Insitutet, Stockholm, Sweden

**Keywords:** Blood alcohol concentration, BAC, Club drugs, "Clubs against Drugs", Cruise ship, Electronic music dance event, EMDE, Illicit drug, Oral fluid drug testing, Saliva

## Abstract

**Background:**

During the last few decades the use of club drugs (e.g., cocaine, amphetamines) has been of increased concern in nightlife settings. Traditionally, surveys have been used to estimate the use of club drugs, however, they mostly rely on self-reports which may not be accurate. Recent advances have allowed for readily accessible drug testing methods such as oral fluid drug testing. Nevertheless, research using oral fluid sampling to measure the frequency of drug use in the club environment is scarce. The objective of this study is to evaluate the feasibility of measuring the frequency of alcohol and drug use among Swedish clubbers using breath alcohol and oral fluid drug testing.

**Method:**

The setting was a 40 hour electronic music dance event (EMDE) on a cruise ship on the Baltic Sea, departing from Sweden, with 875 passengers. Groups of participants at the EMDE were randomly invited to participate. Data were collected with face-to-face and self-administered questionnaires. Further, oral fluid samples were collected to determine illicit drug use, and blood alcohol concentration (BAC) levels were measured using a breath analyzer.

**Results:**

A total of 422 passengers were asked to participate in the study whereof 21 declined (5.0% refusal rate). Of the 401 study participants (accounting for 45.8% of all attendees), 5 declined oral fluid drug testing. Results show that there was a discrepancy between self-reported and actual drug use as 10.1% of the participants were positive on illicit drug use (amphetamines, ecstasy/MDMA, cannabis, cocaine), while only 3.7% of the participants reported drug use during the last 48 hours. The average BAC level was 0.10% and 23.7% had BAC levels ≥ 0.15%, while 5.9% had levels below the detection limit. The mean BAC levels for the illicit drug users were significantly higher (*p *= 0.004) than for non-drug users (0.13% vs. 0.10%). Self-reported AUDIT-C scores (using a threshold of ≥ 5 for men and ≥ 4 for women) revealed that 76.0% of the men and 80.7% of the women had risky alcohol consumption patterns.

**Conclusion:**

This study indicates that it is feasible to conduct breath alcohol and oral fluid drug testing in a Swedish club setting.

## Background

The use of illicit drugs in the nightlife setting is a public health concern since it is associated with violence, drug driving, and risky sexual behavior [1-5]. In Sweden, problems related to club drug use have been attributed to an increased availability of club drugs and a reduction in drug prices [6,7], as well as an increase in the number of licensed premises with extended opening hours [8,9]. In 2002, STAD (Stockholm Prevents Alcohol and Drug Problems) initiated a multi-component community-based club drug prevention program, named "Clubs against Drugs". Program evaluations showed an increase in doormen interventions towards drug-intoxicated patrons (e.g., refused entry into the club) [10,11], a decrease in self-reported drug use among staff at licensed premises, and a decrease in staff's observed drug use among patrons [12]. Nevertheless, club drugs at licensed premises remain a major problem in the Swedish nightlife scene, and we are missing information on actual frequency rates of illicit drug use among patrons.

There are established protocols for measuring patrons' blood alcohol concentration (BAC) in the nightlife setting (e.g., [13-15]). In contrast, similar protocols for measuring illicit drugs have been lacking due to the challenges of collecting biological specimens like blood and urine that have traditionally been used for detection of drugs and their metabolites. However, recent advances in the drug testing field have allowed for the use of alternative specimens such as oral fluid, which now can be used to detect a variety of drugs including amphetamines, cocaine, ecstasy/MDMA, opioids, and cannabis [16-18]. Oral fluid is readily accessible and sampling can be carried out by non-medical personnel without need for special facilities.

Based on these advances, researchers in the U.S. developed a portal methodology to collect both self-reported data and biochemical assays to measure alcohol and illicit drug use among patrons as they entered and exited clubs [19]. Using this methodology, Miller and co-workers reported that one out of four patrons at so called electronic music dance events (EMDEs) was using drugs [20], and the most frequently used illicit drugs were cannabis, cocaine, amphetamine, and ecstasy/MDMA [21]. They further found that about 33% of the patrons had blood alcohol concentrations (BAC) levels of 0.08% or more.

Since the portal methodology has proven to be a useful tool to investigate alcohol and drug use among patrons at EMDEs in the U.S., it was our intent to adopt this method and test it at a similar event in Sweden. However, unlike the U.S. nightlife scene, similar events rarely occur in Sweden today, mainly due to the increased control and sanctions taken by the police authorities as a reaction towards the appearance of the rave culture [22] in Sweden during the 90s. Although EMDEs are rare in Sweden, these events are nowadays arranged on cruise lines that depart from Sweden and take place on international waters where Swedish laws and regulations do not apply and Swedish Authorities have difficulties operating.

Furthermore, while data collected in the U.S. demonstrate the willingness among club-goers to participate in studies involving biological drug testing [21], we had concerns regarding the adoption of a similar methodology in a Swedish setting-especially given the strict enforcement of Swedish narcotic laws. Additionally, the overall population in Sweden is perceived to have more restrictive attitudes towards illicit drug use as compared to the U.S.. For instance, although staff at licensed premises in Stockholm report high levels of drug use, almost 70% agree with Swedish drug laws stating that it should be illegal to be drug-intoxicated [12].

The objective of this study is to evaluate the feasibility of measuring the frequency of alcohol and drug use among Swedish clubbers, using breath alcohol and oral fluid drug testing, in a similar setting as in the aforementioned U.S. studies (i.e., EMDEs). In particular, we are interested in the overall participation rate and the acceptance to give an oral fluid sample. We will also present data on self-reported and measured alcohol and illicit drug use, participants' risky alcohol consumption patterns, and the discrepancy between self-reported and measured drug use. To our knowledge, this is the first study outside of the U.S. using biochemical sampling in a club setting to measure the frequency of patrons' illicit drug use.

## Methods

### Setting

Since large EMDEs in Sweden today are routinely hosted on cruise ships, cruise lines and organizers of EMDEs were approached to gain approval to conduct the study on an EMDE cruise. Permission was obtained to carry out the study at a specific event, however, it was requested that neither the name of the cruise line, the organizer, the EMDE, or the exact date of the event be publicized.

Data collection took place on a 40 hour EMDE cruise departing from Sweden during the spring of 2011. There were a total of 875 paying passengers and approximately 100 additional people involved in the production of the EMDE such as DJs and sound/lightning technicians. The minimum age requirement to participate in the EMDE cruise was 18 years (which is the legal drinking age in Sweden). When boarding the ship, all passengers were required to pass by police officers and drug detecting dogs. In addition, there were a number of police officers present in the departure hall.

All passengers received an event program by the organizers which also contained information about the purpose of the study, that participation was strictly voluntary and confidential (i.e., no personal identifying information was collected), and that participants would receive an incentive in the form of a food coupon worth approximately U.S. $15 or 10€.

On the ship there were several restaurants and bars, and a total of two dance floors. Alcohol could be purchased both at bars/restaurants and at a duty free liquor store selling beer, wine, and hard liquor which could be consumed in the passengers' cabins. During the 40 hour event there were approximately ten security staff that circulated around the ship including the dance floors, bars, and the cabin hallways. The main task of the security staff was to maintain order and prevent unruly and aggressive behavior. In principle, the entire cruise ship was turned into a club, from 10 p.m. and 6 a.m. for two nights, during which time the EMDE attendees moved between different areas on the ship (i.e., the "club area" was not only restricted to the dance floors and bars, and alcohol could be consumed everywhere and at any time).

### Measures

To collect data on demographics and patterns of alcohol and drug use the participants were interviewed using a face-to-face questionnaire and asked to complete an additional self-administered questionnaire. The face-to-face questionnaire contained questions on demographics (e.g., age, gender), alcohol consumption during the present day/night, and the AUDIT-C (Alcohol Use Disorders Identification Test Consumption) instrument [23,24] screening for risky alcohol use. The self-administered questionnaire contained other demographic questions (e.g., marital status, residence, employment) and questions concerning licit and illicit drug use. Substances surveyed included cannabis, cocaine, amphetamines, and ecstasy/MDMA with regards to use ranging from ever use to last 48 hour use, as well as planning to use at this event.

Participant BAC levels were measured using breath analyzers (Lion Alcolmeter™ SD-400, Lion Laboratories Limited, Barry, U.K.) shown to have a high validity [25]. Recent drug use was measured by collecting oral fluid samples (Quantisal™ Saliva Collection Device, Immunalysis Corporation, Pomona, CA, U.S.A.) which have effectively been used to test for illicit drugs [17]. A number of studies have confirmed the validity and reliability of utilizing oral fluids to detect recent use of illicit drugs by comparing urine and/or blood tests with oral fluids [26-29]. Based on previous research, the analysis of the oral fluid samples were limited to the four most commonly used club drugs in Sweden: cannabis, cocaine, amphetamine, and ecstasy/MDMA [12].

### Procedure

On board the cruise ship, a data collection area was allotted by the organizer. This area was centrally located in a passageway nearby the information desk, the duty free liquor store, as well as the two dance floors. A modified portal survey methodology was used, where data were collected once for each participant, instead of twice (upon entering and exiting an event) as described by Miller and co-workers [19,21]. In order to collect entering and exiting data as in the U.S. study, data collection would have to occur when passengers boarded and disembarked the ship. However, boarding and leaving the ship takes place within a limited time-frame (approximately 1.5 hours for boarding and 30 minutes for disembarking), which would have resulted in very few study participants. Further, measuring BAC levels when passengers disembarked, several hours after the EMDE ended, would not have reflected the actual intoxication levels during the event. As a result, we collected data during the EMDE as the attendees moved between the different dance floors, bars, cabins, and the liquor store.

A total of two research supervisors and 12 research staff, divided into four teams (each consisting of three staff members) worked in parallel during the two nights of data collection. Groups, as opposed to individual persons, were randomly invited to participate by a recruiter in each team. The reason to recruit groups was based on previous research indicating that the refusal rate is lower when an entire group is allowed to participate [19]. The recruiter approached the first person passing down the passageway and gave information about the study. If the person belonged to a group, all members of that group were invited to participate.

An informed consent statement was verbally presented to all the participants and a copy of this information was offered. The process of obtaining consent was documented by the research team. Since it was important to maintain confidentiality, no signatures were required. If a whole group, or a person(s) in a group, refused to participate, the recruiter recorded them as drop-outs and noted the gender, approximate age, and asked them why they did not want to participate.

Once the individuals had agreed to participate, they were given a glass of water to rinse their mouths to get accurate readings from alcohol breath test and oral fluid samples. The face-to-face interview was then initiated, followed by the breath test. The result from the breath analyzer was instantly available on site. If requested, the participants were given their BAC level. Next, oral fluid samples were collected from the participants by placing the Quantisal™ collection pad in the mouth. During this process, the participants filled out the self-administered questionnaire. When data collection was completed the participants were given the food coupon. To assure that each participant was only included in the study once during the 40 hour event, the participants were marked with an ultraviolet (UV)-inked stamp. A unique identification number for each participant allowed the pairing of the two questionnaires and oral fluid sample. The oral fluid samples were stored at approximately 2°C throughout the remainder of the cruise. Once the EMDE was completed the oral fluid samples were transported directly to the Pharmacological Laboratory at the Karolinska University Hospital, where they were stored at -20°C pending analysis.

### Oral fluid analysis

The Quantisal™ collection devices with collection pads were thawed and subjected to mild shaking for 1 h at room temperature. Each container was weighed, followed by centrifugation and careful removal of the collection pad. Sample preparation was performed as described by Øiestad et al. [18] with only minor changes: 90 ng/mL delta-9-tetrahydrocannabinol-d3 and 50 ng/mL amphetamine-d5 were added to a saline buffered solution (Extraction Buffer, Immunalysis, Pomona, CA, U.S.A.) to be used as internal standards for delta-9-tetrahydrocannabinol (THC) and alkaline drugs (amphetamine, methamphetamine, 3,4-methylenedioxymethamphetamine (MDMA), cocaine) respectively. After being dried using a stream of nitrogen, samples containing alkaline drugs were redissolved in 55 μL 0.1% formic acid, and THC samples in 15 μL methanol. Thereafter, 40 μL methanol: 0.1% formic acid (50:50) was added to each sample and transferred into screw cap vials. The samples were analyzed using positive electrospray liquid chromatography-tandem mass spectrometry (LC-MS/MS, Waters Quattro Premier), operating in the selected reaction monitoring mode. The applied reporting limit was 1 ng/mL (neat oral fluid) for all analytes.

### Statistical analysis

The SPSS Statistics 19 software (IBM Corporation) was used to analyze the data in terms of descriptive analyses, generating frequency and contingency tables. Here, bivariate analyses yielding Pearson *χ*^2 ^statistics were used to investigate the relationship between variables using a significance level of *p *≤ 0.05. In addition, a *t*-test was used to compare means between independent samples, and Cohen's κ was used as a measure of agreement between self-reported drug use and oral fluid drug testing. Finally, to further investigate the association between variables and control for potential confounders, multivariate logistic regression analyses was performed yielding odds ratios (O.R.) and corresponding 95% confidence intervals (C.I.). Here, the dependent variables were "illicit drug use" (yes vs. no) and "BAC levels" (≥ 0.1% vs. < 0.1%), while the independent variables were "gender", "age" (≥ 25 years vs. < 25 years), "relationship status" (no relationship vs. relationship), "time" (2 a.m. or later vs. before 2 a.m.), frequency of "clubbing" (≥ 1 times/week vs. < 1-2 times/month), overall "risky alcohol consumption" pattern (yes vs. no), as indicated by AUDIT-C using threshold scores of ≥ 5 for men and ≥ 4 for women.

### Ethics statement

This study has been approved by the Regional Ethical Review Board at the Karolinska Institutet in Stockholm (Registration number: 2009/797-31/4).

## Results

The purpose of this study was to evaluate the feasibility of using breath alcohol and oral fluid drug testing to measure the frequency of alcohol and drug use among Swedish clubbers. Overall, data collection proceeded according to plans. The two research supervisors and the four research teams worked from 10 p.m. (4 hours after departure) in the evening until 6 a.m. in the morning. Throughout the EMDE, the teams were fully occupied collecting data from the participants; as soon as one group of participants had completed data collection, a new group of participants were recruited. A total of 422 passengers (belonging to 238 groups having a mean group size (± S.D.) of 1.73 ± 0.97) were asked to participate in the study whereof 21 (5.0%, belonging to 12 groups, mean size 2.70 ± 1.16) declined, resulting in 401 participants (36.2% females). Thus, 45.8% of the 875 paying passengers were included in the study. During the first night of the EMDE, data were collected from 206 attendees (51.4% of the whole study sample), and on the second night an additional 195 attendees (48.6%) were included in the study. About half of the participants (44.9%) took part in the study before 2 a.m. and the other half after 2 a.m.. On average, each of the four research teams collected data from about six participants per hour. Missing data on specific items varied but never exceeded 7.2% (which concerned a question whether or not the participants were planning to use illicit drugs at this event). A total of five participants declined to give saliva samples. It is worth mentioning, that in some cases (< 5 participants) the saliva collection procedure was overly time consuming (> 15 min) due to dryness of mouth, and a number of participants reported that their low saliva production was related to medications (e.g., anti-depressants).

### Demographics

The overall characteristics of the participating patrons are presented in Table 1. The mean age of the participants was 25.7 ± 6.0 years (26.5 ± 6.2 and 24.2 ± 5.2 for men and women, respectively) ranging from 18 to 45 years. The proportion of students was approximately 30%. Of these, about 25% were high school students and about 60% were studying at university level. The participants were from various regions in Sweden: 40.1% from one of the three largest cities (Stockholm, Gothenburg, and Malmoe), 55.1% from minor cities or rural areas, and 4.7% from abroad. Almost half of the participants (48.5%) reported going out clubbing 1-2 times per month, while 32.0% reported clubbing ≥ 1 times per week, and 19.2% reported clubbing rarely.

**Table 1:** Overall demographics and characteristics of patrons

Characteristic	Females (n = 145) % (n)	Males (n = 256) % (n)	Total (n = 401) % (n)
Age			

< 25 years	62.8 (91)	48.8 (125)	54.0 (216)

25-29	22.1 (32)	21.5 (55)	21.7 (87)

30-39	13.8 (20)	25.8 (66)	21.4 (86)

40+	1.4 (2)	3.9 (10)	3.0 (12)

Employment/student status			

Employed, full time	28.2 (40)	65.5 (163)	51.9 (203)

Employed, part time	31.0 (44)	12.0 (30)	18.9 (74)

Unemployed	14.1 (20)	4.8 (12)	8.2 (32)

Student, full time	31.7 (45)	19.9 (50)	24.2 (95)

Student, part time	7.0 (10)	6.0 (15)	6.4 (25)

Nonstudent	59.9 (85)	70.9 (178)	66.9 (263)

Relationship status			

In a relationship	53.8 (78)	42.5 (108)	46.7 (186)

Not in a relationship	44.8 (65)	55.9 (142)	51.9 (207)

### Illicit drug use

**Figure 1:**
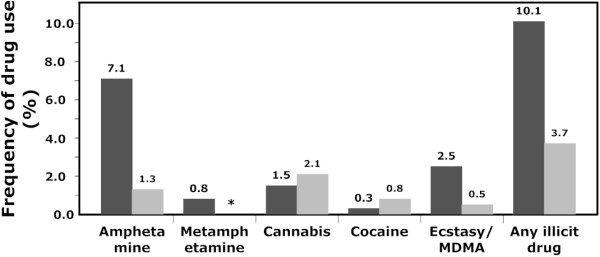
**Frequency of illicit drug use**. A comparison between the frequency (in per cent) of illicit drug use as determined by oral fluid drug testing (dark grey, n = 396) compared to self-reported drug use within the past 48 hours (light grey, n = 382). Since questions about metamphetamine use were not included in the self-report questionnaire, a subsequent estimate was not available (asterisk).

Self-reported ever use of drugs among the participants was 49.1% and 9.2% reported that they usually use drugs when clubbing. Further, 3.7% reported having used drugs during the last 48 hours, and 6.0% were planning to use drugs during the EMDE (Table 2 and Figure 1). The most commonly self-reported drug during the last 48 hours was cannabis, followed by amphetamine, cocaine, and ecstasy/MDMA. A total of 396 saliva samples were collected and analyzed. Analysis revealed that 10.1% of the participants tested positive for at least one illicit drug (20.0% of the drug users were polydrug users). The most commonly used drugs were amphetamines, followed by ecstasy/MDMA, cannabis, and cocaine. The mean values (ng/mL) and ranges were: amphetamine, 1587 (2-32352); methamphetamine, 725 (15-1880); MDMA, 930 (43-7288); cocaine, 11224; THC, 255 (58-297). Of the 40 illicit drug users the majority were males (75.0%). The frequency of any illicit drug use was 11.8% for men and 6.9% for women (Table 3), however, this difference is non-significant. Both men and women had the same drug preference but none of the women tested positive for cocaine or metamphetamine. Of those who tested positive for drug use over the course of the two evenings, a slightly higher number (57.5%) were found during the second night of data collection. There were a larger (although non-significant) proportion of participants (62.5%) who were positive on drugs later in the night (2 a.m. or later) compared to earlier (before 2 a.m.).

**Table 2:** Patrons self-reported drug use (%)

	Have tried	A couple of times/month	Every weekend	A couple of times/week	Last 48 hrs	Usually use when clubbing	Planning to use at this event
Cannabis	41.5	7.2	2.6	1.5	2.1	6.1	1.9

Amphetamines	18.5	2.3	0.3	0.0	1.3	3.7	1.9

Cocaine	12.3	0.8	0.0	0.3	0.8	1.6	0.8

Ecstasy/MDMA	16.2	0.3	0.0	0.0	0.5	1.8	0.8

**Table 3:** Frequency of actual illicit drug use for males and females (%)

	Amphet amine	Metamph etamine	Cannabis	Cocaine	Ecstasy/MDMA	Any illicit drug
Male	8.6	1.2	2.0	0.4	2.4	11.8

Female	4.2	0	0.7	0	2.8	6.9

There is a poor agreement (Cohen's κ = 0.198, *p *= 0.001) between self-reported and actual drug use since only 16.7% of those who tested positive for any drug (using oral fluid drug testing) reported having used drugs during the last 48 hours. Furthermore, of those who tested negative for any drugs, 2.3% reported that they had used drugs during the last 48 hours.

### Alcohol use

**Figure 2:**
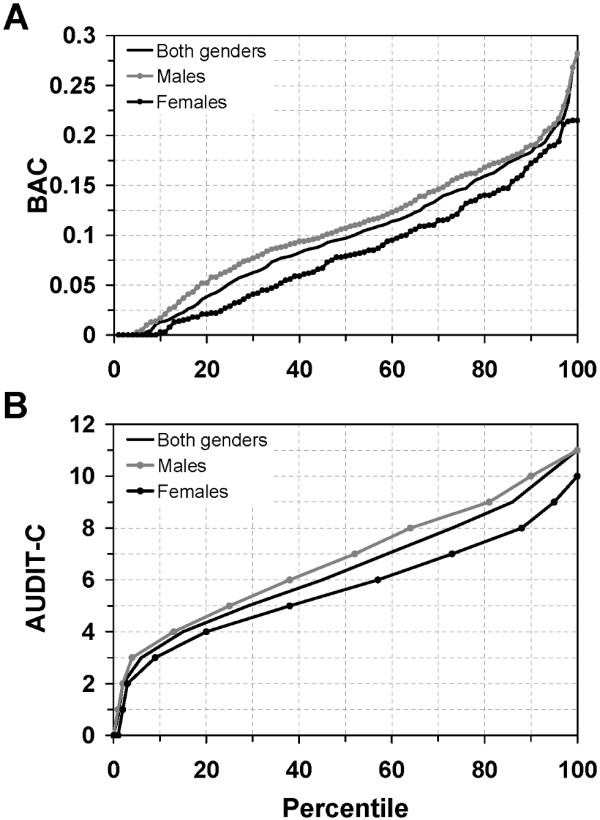
**Measured blood alcohol concentrations and AUDIT-C scores**. (**A**) Blood alcohol concentrations (BAC) for males (n = 250, grey filled circles), females (n = 142, black filled circles), and both genders (n = 392, solid line) distributed over the percentile range. (**B**) Total AUDIT-C scores (ranging from 0-12 points) for males (n = 254, grey filled circles), females (n = 145, black filled circles), and both genders (n = 399, solid line) distributed over the percentile range.

The mean number of self-reported standard drinks (defined as drinks containing 12 grams of pure alcohol, e.g., one bottle of beer, one glass of wine) consumed by the participating patrons each evening/night was 8.8 (± 5.7) drinks (9.9 ± 6.2 and 6.8 ± 4.0 for men and women, respectively). The average BAC level was 0.10% (± 0.06, range 0.00-0.28); for men the mean value was 0.11% (± 0.06) and for women 0.08% (± 0.06). As seen in Figure 2A, about 62% of the participating patrons (70% for men, 49% for women) had a BAC level of ≥ 0.08%, while approximately 23% (28% for men, 14% for women) had a measured level of ≥ 0.15%, and almost 6% (7% for men, 3% for women) had a level of ≥ 0.20%. About 6% (4% for men, 9% for women) of the participants had not consumed any detectable levels of alcohol. There was no difference concerning measured BAC levels between the first and second night of data collection, nor before or after 2 a.m.. However, there are significant differences between males and females as 54.4%, and 37.3% of men and women, respectively, had a BAC level of 0.10% or more (*χ*^2 ^= 10.58, d.f. = 1, *p *= 0.001). Other factors that were significantly associated to participants having a BAC level of 0.10% or more was illicit drug use, an overall risky alcohol consumption pattern, and frequency of clubbing (Table 4). These associations remained after controlling for possible confounding factors using multivariate analyses.

**Table 4:** Results from bivariate and multivariate analyses using logistic regression modeling to adjust for possible confounders

Predictor variables	Bivariate analyses			Multivariate analyses		
	
	χ^2 ^(d.f.)	**O.R**.	**95% C.I**.	χ^2 ^(d.f.)^a^	**O.R**.	**95% C.I**.
*D.V.: Illicit drug use (yes vs. no)^b^*						

BAC level (≥ 0.1% *vs*. < 0.1%)	7.66(1)**	2.65	1.30-5.40	5.63(1)*	2.53	1.18-5.43

Risky alcohol consumption (yes *vs*. no)	2.47(1)	2.14	0.81-5.63	1.84(1)	2.17	0.71-6.61

Gender (males vs. females)	2.40(1)	1.79	0.85-3.78	1.35(1)	1.59	0.72-3.45

Clubbing (≥ 1 times/week *vs*. < 1-2 times/month)	1.22(1)	1.46	0.75-2.85	0.21(1)	1.18	0.57-2.46

Time (2 a.m. or later *vs*. before 2 a.m.)	2.76(1)	1.79	0.89-3.58	2.88(1)	1.89	0.91-3.94

Age (≥ 25 years *vs*. < 25 years)	0.02(1)	0.95	0.49-1.83	0.34(1)	0.81	0.40-1.36

Relationship status (no relationship *vs*. relationship)	0.71(1)	0.75	0.39-1.46	2.32(1)	0.58	0.28-1.17

*D.V.: BAC level (≥ 0.1% vs. < 0.1%)*						

Illicit drug use (yes *vs*. no)	7.66(1)**	2.65	1.30-5.40	5.79(1)*	2.55	1.19-5.45

Risky alcohol consumption (yes vs. no)	13.51(1)***	2.57	1.54-4.30	8.50(1)**	2.28	1.31-3.96

Gender (males vs. females)	10.58(1)**	2.00	1.31-3.05	7.49(1)**	1.88	1.19-2.94

Clubbing (≥ 1 times/week *vs*. < 1-2 times/month)	11.83(1)**	2.11	1.38-3.25	4.05(1)*	1.62	1.01-2.60

Time (2 a.m. or later *vs*. before 2 a.m.)	0.96(1)	1.22	0.82-1.82	0.28(1)	1.12	0.73-1.72

Age (≥ 25 years *vs*. < 25 years)	0.54(1)	1.05	0.71-1.56	0.89(1)	1.24	0.80-1.92

Relationship status (no relationship *vs*. relationship)	3.06(1)	1.43	0.96-2.14	0.67(1)	1.20	0.77-1.87

^a^Wald *χ*^2 ^statistics based on logistic regression modeling

^b^*DV *is the dependent variable

*p < 0.05

**p < 0.01

***p < 0.001

Using an AUDIT-C threshold commonly used in European countries (i.e., ≥ 4 for women and ≥ 5 for men) reveals that 76.0% of the men and 80.7% of the women (77.7% for both genders) had self-reported risky alcohol consumption patterns (Figure 2B). This proportion increases to 87.8% and 92.4% for men and women (89.5% for both genders), respectively, using a threshold commonly used in the U.S. (i.e., ≥ 3 for women and ≥ 4 for men). These gender differences with regards to risky alcohol consumption are not significant.

### Alcohol and illicit drug use

A greater proportion (although not significant) of the illicit drug users had self-reported risky alcohol consumption according to the AUDIT-C scores compared to non-drug users (87.5% vs. 76.6%). All the illicit drug users had been drinking alcohol. The mean BAC levels for the illicit drug users were significantly higher than for non-drug users, 0.13% compared to 0.10% (t = 2.893, df = 390, *p *= 0.004). The significant association between BAC levels and illicit drug use remained after controlling for possible confounding factors as seen in Table 4 (*p *= 0.02).

## Discussion

These findings indicate that it is feasible to measure the frequency of alcohol and drug use among Swedish clubbers using breath alcohol and oral fluid drug testing. Previous studies using breath analyzers to measure BAC levels among patrons have successfully been conducted in a Swedish nightlife setting [30,31], but oral fluid drug testing has never been used to measure illicit drug use. During the planning phase of this study, concerns were raised regarding the possibility of using biochemical markers to measure illicit drug use among Swedish clubbers. This was mainly due to potential cultural differences between countries concerning illicit drug use. For instance, showing obvious signs of illegal drug-intoxication is perceived to be more stigmatized in Sweden as compared to the U.S.. This would impede studying illicit drug use among Swedish clubbers as they might be less inclined to take part in this type of study. Nevertheless, the data collection procedure went according to plans and we were surprised by the amount of positive feedback from the EMDE attendees as well as the management and staff at the cruise ship. In fact, we were invited by the cruise line management to contact them if we wanted to conduct further studies. The research teams' recruiters were able to approach nearly half of the EMDE attendees and 95.0% agreed to participate which is a considerable higher participation rate than the 56.0% reported in the U.S. study [21].

There are several possible factors that may have influenced the high participation rate: (i) the recruitment process was crucial to the study and appropriate staff (same age group as the EMDE attendees, outgoing personality, and socially skilled) were carefully selected and trained, (ii) the incentive (food coupon corresponding to U.S. $15 or 10€) appeared to be valuable for this target group, (iii) the oral fluid sample device was easy to administer, well accepted by the participants, and did not give results on site, (iv) the EMDE organizers gave written support to our study in the event program, and finally, (v) the participants showed great interest in finding out their BAC levels. In fact, not only were we interested in measuring the participant's actual BAC levels, our thought was that the inclusion of an initial BAC test in the data collection process would make the oral fluid testing for illicit drug use less dramatic.

Almost all participants, 94.1% had been drinking alcohol and 10.1% tested positive for illicit drug use. From an international perspective the frequency for illicit drug use might seem low. In comparison, at the U.S. EMDEs an average of 26.0% of the attendees were drug-positive when leaving the events [21]. There are several potential explanations for the observed lower levels of illicit drugs among the Swedish clubbers. It is possible that the lower levels might reflect the actual drug use among the patrons at this event. EMDE patrons in Sweden might use illicit drugs to a lesser extent than for example their counterparts in the U.S.. However, as in many other countries EMDEs in Sweden have been associated with high frequency of illicit club drug use. More specifically, EMDEs on cruise ships in Sweden have previously had problems with illicit club drugs. During an EMDE cruise in 2005, the police authority (working undercover on the cruise) estimated that about half of the patrons were under the influence of illicit drugs and over 30 patrons were arrested (when leaving the cruise ship) for drug crimes, and cocaine, amphetamines, and ecstasy/MDMA were confiscated. As a result, preventive measures have been taken by the cruise lines, police authorities, and the customs control department, which could have resulted in decreased amounts of drugs brought on to the cruise ship. For example, the fact that all passengers had to pass by the police's drug detecting dogs when boarding, along with the police authority present in the departure hall could explain the comparatively lower observed illicit drug rates. In fact, at this cruise the police authority reported about 20 drug-related crimes at the point of boarding the ship. It could also be the case that the patrons that used drugs during the cruise were underrepresented in our sample. Even though 45.8% of the patrons were included in the study and the refusal rate was low, the patrons that used drugs could have avoided walking by the data collection area.

There is a discrepancy between self-reported drug use and the results from the oral fluid tests as only 3.7% of the participants reported having used drugs during the last 48 hours, while 10.1% tested positive for drug use (Figure 1). Even though self-reported illicit drug use was confidential, only 16.7% of the drug users reported having used any drug during the last 48 hours. This type of underreporting of self-reported drug use has also been observed previously, and could possibly be explained by reporting bias as people may be reluctant to report illicit drug use. It could also be the case that some participants were unaware of drug intake. Nevertheless, the agreement between self-reported and actual drug use was poorer in our study (Cohen's κ = 0.198, *p *= 0.001) compared to what has been reported by Johnson and co-workers (Cohen's κ = 0.53, *p *< 0.01) [32]. Furthermore, 2.3% of those with negative drug tests had reported using illicit drugs during the last 48 hours. This could be explained by a person believing they have ingested a specific drug while they have ingested another substance. Drugs can be detected in oral fluids for up to three days and depends on a variety of factors such as the physiochemical properties of specific drugs, the strength (i.e., drugs may be diluted) and the amount of a drug taken, and the route of administration. In addition, the metabolism of drugs varies from person to person and depends on factors like age, body mass, liver function, and genetic factors [29,33]. The frequency of positive drug tests/self-reported drug use was low for some drugs, precluding comparative analysis between the drugs. However, one could speculate that the greater discrepancy between self-reported drug use and oral fluid drug test found for amphetamine and ecstasy/MDMA is due to a less acceptance of these drugs as compared to cannabis and cocaine (considered by some people to be a softer drug, and a more glamorous drug, respectively).

The BAC levels found in this study (mean value 0.1%) were high compared to other studies conducted at university student pubs in Sweden (mean values between 0.075-0.087%) [30]. At the U.S. EMDEs, 33% of the patrons had BAC levels ≥ 0.08%, while in our sample this figure corresponded to 62% [21]. One reason for the observed high BAC levels is probably the high availability and low control of the large quantities of alcohol that could be purchased in the duty free shop and consumed in the cabins at any time during the EMDE. In contrast, regular clubs in Sweden are covered by Swedish alcohol laws, stating that obviously intoxicated patrons should not be served alcohol or even be denied entry into licensed premises. Another reason could be related to the event taking place on a cruise ship where EMDE attendees only needed to find their way back to their cabins (i.e., attendees did not have to be concerned about home transportation).

The participants' high BAC levels measured in this study were significantly associated with frequent clubbing and an overall (i.e. not related to this event) risky alcohol consumption pattern as indicated by the AUDIT-C scores (77.7% and 89.5% had risky alcohol consumption using European and U.S. threshold scores, respectively) (Table 4). In other words, it may be the case that problem drinkers are attracted to this type of event. However, the vast majority (80.4%) of our sample reported going clubbing at least 1-2 times per month which means that these participants attend land-based clubs on a regular basis. Furthermore, all the illicit drug users had been drinking alcohol and even had significantly higher BAC levels relative the non-illicit drug users. The association between illicit drug use and a high BAC level remained even after controlling for other possible confounders using multivariate analyses (Table 4). Therefore, the common perception that clubbers who use illicit drugs drink less alcohol than non-illicit drug users was not supported by the results from this study.

There are a few limitations to this study worth mentioning. We were allotted a data collection area on the cruise ship, that the EMDE attendees did not have to pass by, and it could be the case that drug-intoxicated attendees avoided our research team. One might also question the accuracy in self-reports by highly intoxicated people. In this study, 23% had BAC levels ≥ 0.15% which might have affected cognitive functioning, memory, as well as judgment; all contributing to lower capability of filling out a questionnaire. This study was conducted during a particular EMDE at a cruise ship, which can be regarded as a special type of club environment. Moreover, when boarding the ship, there were drug detecting dogs and police officers present which may have affected the frequency of drug use. For instance, if this study had been conducted at clubs taking place at licensed premises located in a city, it is possible that our results would have been different (e.g., higher refusal rates and different frequency rates). An important challenge when conducting a portal survey at regular clubs concerns the recruiting process as people do not want to waste any time participating in research, since they are eager to get into the event or quickly leave. At regular clubs people can arrive and leave whenever they want, whereas passengers on a cruise ship have nowhere else to go. In order to gain a better understanding of drug use at EMDEs we would have to include a broader range of venues as well as a greater number of events. Further, less commonly used drugs such as GHB (gamma-hydroxybutyric acid), mushrooms, and benzodiazepines could be added to the analysis of the oral fluid samples.

Traditionally, the majority of alcohol and drug prevalence studies rely on surveys, however, our study highlights the importance of using biochemical markers to properly estimate drug use in this type of setting. The methodology used in this study provides the opportunity to combine and measure the actual BAC levels and illicit drug use. Not only can this method be used to follow drug trends in settings such as the club scene, sport events, and festivals, it can also be used to increase the awareness of the club drug problem in communities, mobilize stakeholders, and evaluate the effects of various drug prevention programs. Furthermore, studies have shown that young adults are a high risk-group for drug and alcohol-related problems and this study suggest that one arena where this age group can be reached with preventive measures is at EMDEs. It is important to find settings where emerging adults can be targeted with alcohol and drug prevention programs such as Responsible Beverage Service programs [34-36], and club drug environmental strategies [10,12].

## Conclusions

The purpose of this study was to test the feasibility of measuring the frequency of alcohol and drug use in a Swedish club setting using breath and oral fluid analysis. The vast majority (95.0%) of the approached EMDE attendees agreed to participate in the study, whereof only five attendees declined the oral fluid drug testing. We found that about one out of ten participants were positive on illicit drug use and the average BAC level was 0.10%. The illicit drug users had significantly higher mean BAC levels than the non-drug users. We conclude that it was feasible to use alcohol breath and oral fluid drug testing at this EMDE. However, to what extent this method can be used in other types of settings needs to be explored in future studies. Nevertheless, we believe that this methodology, combining surveys with biochemical markers, provides a useful and more accurate tool to measure actual drug and alcohol use in the nightlife scene as well as other types of settings.

## Competing interests

The authors declare that they have no competing interests.

## Authors' contributions

EW, JGA, SA, and THE planned and designed the experiments. JGA and THE were responsible for and performed the data collection. MS contributed to data collection. JGA and THE analyzed the data and interpreted the results. EW and SA contributed to the interpretation of results. MS and OB analyzed the oral fluid samples. JGA and THE wrote the paper. All authors commented on and approved the final manuscript.
